# Increasing Latino Parents’ Verbal Interactions with Their Preschool-Aged Children

**DOI:** 10.5402/2012/652406

**Published:** 2012-03-07

**Authors:** Sabina Gesell, Dan Wallace, Tommaso Tempesti, Vanessa Hux, Shari Barkin

**Affiliations:** 1 Vanderbilt School of Medicine, 2nd Floor, 2146 Belcourt Avenue, Nashville, TN 37212, USA; 2 Vanderbilt University’s Peabody College, 230 Appleton Place, Nashville, TN 37203, USA; 3 University of Massachusetts Lowell, One University Avenue, Lowell, MA 01854, USA; 4 Vanderbilt University School of Medicine, 2200 Children’s Way, DOT8246, Nashville, TN 37232, USA

## Abstract

The rapidly growing Hispanic American population is experiencing an academic achievement gap that seems to be rooted in disparities in early childhood education and literacy development. Children of non-English-speaking immigrant parents are at greatest risk of poor school performance, but there is potential to capitalize on immigrants’ drive by encouraging them to engage with their children in dialog while reading native-language storybooks. This paper reports on a community-based randomized controlled trial (*N* = 79) delivered to mostly Mexican immigrant parents of preschool-age children. Intervention group parents attended three monthly 60-minute sessions based on the Dialogic Reading Model—C.A.R. (Comment and Wait, Ask Questions and Wait, and Respond by Adding More), which teaches parents to have a conversation about pictures in books, with the goal of enhancing verbal exchanges with the child in the parent’s native language. After the 3-month intervention, parents in the bilingual early language development intervention reported placing greater value on children’s active verbal participation in reading compared to control group parents who participated in a healthy lifestyle intervention. These results suggest that Hispanics’ educational outcomes may be improved by educating parents on the value of playful conversations with young children while reading books in one’s native language.

## 1. Introduction

Immigration from Latin America has drastically altered the demographic makeup of the United States in recent years. According to the U.S. Census Bureau, as of July 1, 2006, Hispanics made up 14.8 percent of the population of the United States, a total of 44.3 million people. Even more striking is the fact that, between 2000 and 2006, Hispanics accounted for one half of the growth of the entire US population. Such rapid change has led to an intense examination of many American institutions, especially the education system, which is already burdened by overcrowding and a lack of resources.

Inevitably, the discussion of immigration and schools has raised many questions about how to respond to the needs of a growing and increasingly diverse group of students. Currently, nearly one out of every five students in the US is Hispanic, and when coupled with the fact that 21 percent of those students fail to graduate from high school—a rate four times that of their white peers—the need to make improvements demands even more immediate attention [1]. This statistic has prompted many questions and the need for a solution to addressing the “consistent pattern of underachievement” shown by Hispanic student with respect to educational attainment [2].

Although this achievement gap is clear by the time students reach high school, according to most research, early childhood is the source of the problem. Lahaie found, for example, that children of immigrants with both parents speaking a language other than English in the home were 81 percent more likely to fail to demonstrate English proficiency by the time they entered school [3], and additional evidence has even shown a positive relationship between limited English language proficiency and externalizing symptoms—such as aggression, delinquency, and hyperactivity—as early as the third grade [4]. De Feyter also demonstrated the disparity in cognitive and linguistic ability between children in immigrant and nonimmigrant families and suggested that these differences might be related to the greater familiarity of native-born parents with early education programs and resources [5]. Additional research supports this claim. The U.S. Department of Education found that Latinos were less likely than children from any other racial or ethnic group to be enrolled in an early childhood program [6], and data from the Early Childhood Longitudinal Study (ECLS)—a nationally representative sample—shows that only 47% of children of immigrants attended center-based care prior to kindergarten, compared with 63% of children of the native born [3].

Given these challenges and the fact that children who enter school at a disadvantage often have more difficulty making up ground later on [7], De Feyter argues that increased focus on students’ developmental progress prior to entering school is “the first step … in closing the achievement gap” [5]. Not surprisingly, a large part of this increased focus will mean engaging parents in their children’s education to ensure future success. Studies have demonstrated time and again that parental involvement is one of the keys to improving educational outcomes for students and that it can make an even greater difference for Latinos and other immigrant groups [2, 3, 8–10]. According to data from the ECLS, parental involvement is associated with increases in both English language proficiency and math scores for children of immigrants [3]. In fact, when looking at cognitive learning in the home—which includes literacy activities such as reading books or telling stories—the same data set showed that *each additional unit increase* in children’s books in the home was associated with a 2-percent increase in the odds of children of immigrants being proficient in English, along with a similar increase in math performance.

Although evidence for the connection between parental involvement and academic achievement is overwhelming, the fact remains that many Latino and immigrant parents face barriers to involvement, such as lower levels of English language proficiency, less flexible and more time-consuming work schedules, and less access to transportation. Ultimately they demonstrate lower levels of involvement with their children than native-born parents [2, 11, 12]. Lower English language proficiency can inhibit parents from performing a number of important activities, such as attending school events and parent-teacher conferences, helping with home-work, and reading to their children. Additionally, many parents reported long and demanding work hours and transportation issues as other barriers to involvement [12].

Still, contrary to what many infer from the reduced involvement in this group, studies show that Latino immigrant parents—many of whom have low levels of education themselves—place a great deal of emphasis on the value of education and have very ambitious hopes for the academic achievement of their children. The idea of the “immigrant advantage”—that despite possessing characteristics that typically correlate with academic failure, immigrants’ resilience and drive can provide a path to success—suggests that this group is particularly well suited for cognitive and verbal intervention programs at the early childhood stage [5]. Several studies have been performed in this area; however, very few offer insight into parent-implemented—as opposed to teacher-implemented—interventions among Latinos. Schaller et al. noted this ambition, present in many low-educational, low-income Mexican immigrant mothers, and argued that “The best way to capitalize on immigrant parents’ educational drive for their children is to partner with them … by showing them how their participation in their children’s learning through concrete activities (such as daily reading) may increase their chances of achieving academic success” [13].

One such concrete activity is to promote parental involvement in literacy habits for their children using their native language, such as increasing the extent to which parents engaging in dialog with a child over books and understand that such verbal participation influences language development. By augmenting home-language resources with storybooks and a program to support and encourage home storybook reading, children’s language development may be modified. This is further supported by Mushi’s finding that the more immigrant parents participated in a joint activity with their children, the more the children engaged in linguistic behavior [14]. The majority of immigrant parents, regardless of the level of education, display willingness to participate in their child’s education but did not necessarily know the steps to take [15].

Further needed are strength-based approaches: while researchers know that lower English language proficiency need not be a barrier to academic success and that bilingualism has great advantages, immigrant parents do not typically see fluency in their native language or their child’s bilingualism as strengths. Typically underappreciated by immigrant parents is the fact that their fluency in their native language is not only sufficient, but actually bestows increased advantages to their child compared to monolingual children. Educational experience in the first language does not compromise second-language learning. In fact, bilingual children have been shown to have greater metalinguistic understanding, more cognitive flexibility, better inhibitory control, and greater analogical reasoning skills than their monolingual counterparts. Lou provided evidence of enhanced executive control for both bilingual groups, compared with monolingual (inhibitory, attention, self-control, etc.) [16].

Bilingualism can play a significant role in academic achievement. Lisette found that language proficiency in either the first or second language at school entry predicted academic achievement and language development in the second language [17]. Specifically, it has been shown that there is a positive impact of parental involvement in literacy in one’s native language on school readiness and success. Roberts found that primary-language storybook reading in the home was as effective as home storybook reading in English for promoting English vocabulary acquisition in preschool students who are learning English; children who received storybooks in their primary language performed significantly better on English recognition of target storybook words than English language learning students who read books at home in English [18].

Thus, this study advances the research in this area by investigating the potential for a brief community-based intervention to increase Latino parents’ willingness to engage in dialog with their preschool children over books using their native language.

## 2. Methods

### 2.1. Study Sample.

We implemented a community-based, family-centered, healthy lifestyle randomized controled trial (RCT), *Salud con la familia* (Health with the Family), with an alternative early childhood literacy intervention. Both treatment arms were designed for Latino families with preschool-aged children and were conducted in a public community center guided by principles of community-based participatory research (CBPR). CBPR has evolved over the last decade as an important collaborative approach to research that equitably involves community members who are affected by the issue being studied in all phases of the research process [19]. In our formative research phase, we learned through focus groups that the community would be particularly receptive to a school readiness intervention and thus presented our early language development intervention within that framework. The local Parks and Recreation Department and branch library were our community partners in this study.

The community center was located in a neighborhood characterized as having a relatively high Latino concentration and low socioeconomic status. Inclusion criteria for study participants were the following: (1) self-defined Latino/a, (2) with a child aged 2–6 years who was not currently enrolled in kindergarten or any other healthy lifestyle program, (3) having a valid phone number, and (4) planning on remaining in the city for the next six months. Prospective study participants underwent a 30-minute oral consent process in Spanish before providing written consent for themselves and their preschool-aged child. Recruitment occurred between October 2008 and February 2009. The intervention occurred between March and June 2009. The study was approved by the Vanderbilt University Institutional Review Board (IRB no. 080673).

**Figure 1:**
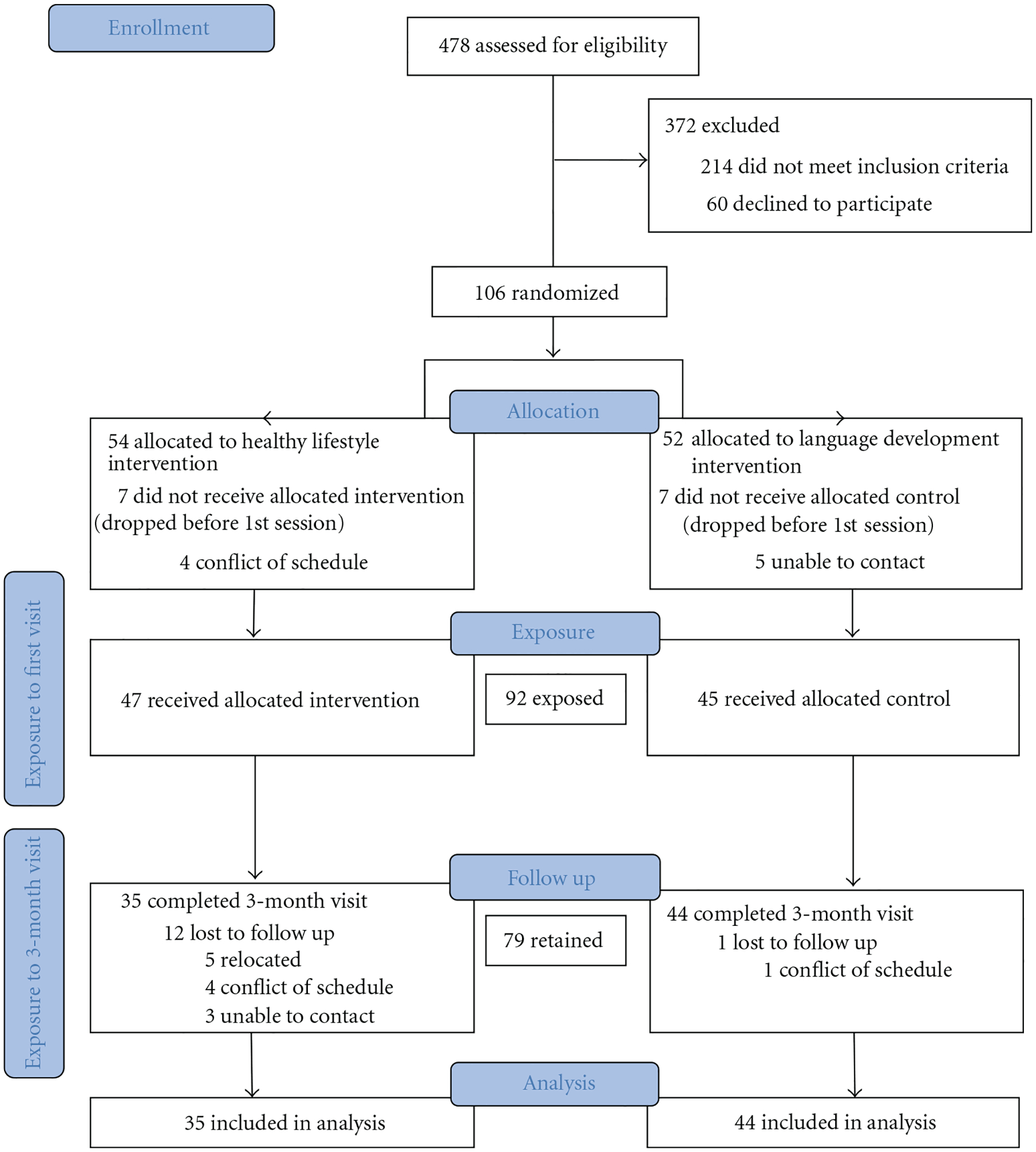
Flow of participants through the trial.

Study randomization occurred after baseline data collection. A total of 106 parent-child dyads were randomized, 92 had exposure to the allocated interventions and completed baseline data collection, 79 were retained and provided data at the end of the intervention period, 3 months later, resulting in an 86% retention rate for those who attended the first session of the assigned interventions (refer to Figure 1). Both transportation to and from study sessions and on-site childcare services were provided free of charge to all study participants to overcome the most frequently cited barriers to study participation [20–23].

### 2.2. Study Design

#### 2.2.1. Bilingual Early Language Development Intervention Group.

We developed an early childhood literacy program following evidence-based recommendation to partner with immigrant parents “by showing them how their participation in their children’s learning through concrete activities (such as daily reading) may increase their chances of achieving academic success” [13] and supplementing home-language resources with storybooks and a program to support and encourage home storybook reading [14]. The bilingual early language development intervention was designed to build school readiness in preschoolers through increased parental verbal participation in Spanish (e.g., built parental skills and knowledge around daily reading, playing word games, how to talk to children, turning off the television) drawn from sections of a tested curriculum, *Language is The Key* [24]. Participants met three times for 60 minutes over the 12-week study period. These sessions were based on the curriculum’s core strategy, the Dialogic Reading Model—C.A.R. (Comment and Wait, Ask Questions and Wait, and Respond by Adding More), which teaches caregivers language facilitation techniques around picture book interactions between parents and their children [24]. This model lends itself to all spanish-speaking parents—importantly including those with low literacy or low-English language skills as its effectiveness in parent’s learning to use these techniques and increasing their children’s language production has been documented in both English [25] and non-English speaking parent-child dyads [26]. Using this reading method, parents learned to have a conversation about the pictures in the book, with the goal of enhancing verbal exchanges with the child in the parent’s native language. The curriculum was enhanced by including group discussions on the unique advantages of immigrant parents’ existing strengths and skills, a tour of the local branch library in spanish that highlighted spanish-language resources in the city-wide library system, building skills to access them, assistance with obtaining a library card, and the provision of three new paperback picture books in spanish.

#### 2.2.2. Healthy Lifestyle Intervention Group.

The healthy lifestyle intervention group served as the comparison group. We made the plausible assumption that the healthy lifestyle intervention of the original study did not affect our focal outcome measure (i.e., parental verbal participation). The healthy lifestyle intervention consisted of a series of 12 weekly (90 min) group sessions on physical activity, nutrition, and energy balance. Every session included interactive group processes around concrete tasks relevant to the purpose of each session (such as building skills to be active with their child, to choose appropriate portion sizes). These group sessions were based on materials designed by the National Latino Children’s Institute (NLCI), which were endorsed by the American Dietetic Association, and customized by the study team to meet the needs and preferences of the local Latino population.

#### 2.2.3. Treatment Fidelity.

Prior to study initiation, a treatment fidelity plan was devised to monitor and enhance the reliability and validity of our behavioral intervention following the methodological practices suggested by the Treatment Fidelity Workgroup of the NIH Behavior Change Consortium [27]. The plan included implementer training and supervision, identification of essential treatment components for verification, sampling to ensure treatment consistency, control for differences between interventionists, and use of fidelity measures (e.g., length, number, frequency of session and participation rates). A study team member observed three sessions of each condition and determined that 100% of the intended key messages were fully discussed, all planned activities occurred, and intervention content was never delivered during control sessions or vice versa.

#### 2.2.4. Randomization.

Participants were randomized in equal proportion to each treatment group using a computer-generated permuted block randomization scheme, with blocks of size 10 to ensure a balance in treatment allocation when the total sample size was reached. A biostatistician generated the randomization list and placed the treatment assignments into nontransparent envelopes, which were sealed and numbered consecutively. Upon giving informed consent, participants received from the research coordinator the next numbered allocation envelope which was opened by the participant. The treatment assignment and number listed on the envelope were recorded by the research coordinator and the participant. Neither researchers nor participants were blinded to participant group allocation.

### 2.3. Data Collection.

All data were collected by trained bilingual data collectors at the community center. Survey questions were read out loud in Spanish in a group format, and individuals marked their responses on the paper survey. The dependent variable of interest was change in parents’ beliefs in the value of reading with preschool aged children and encouraging them to be active participants in the reading process as an upstream indicator of language development.

### 2.4. Measures

#### 2.4.1. Verbal Participation.

The Verbal Participation subscale of the Parent Reading Belief Inventory (PRBI) [28] was selected because the sessions were guided by the Dialogic Reading Model as described previously. The full PRBI scale was developed to examine parents’ beliefs about goals and process of reading aloud to young children and theories of emergent literacy and environmental influence on language development. This scale has been previously validated in Mexican-American parents who averaged 11.12 (SD = 1.62) years of formal education [29]. The verbal participation subscale assesses the value placed on children’s active verbal participation when reading. The nine items are rated on a 4-point Likert scale, ranging from 1 = Strongly Disagree, 2 = Disagree, 3 = Agree, to 4 = Strongly Agree. Sample items are “Reading helps children be better talkers and better listeners; I ask my child a lot of questions when we read; I find it boring or difficult to read to my child (reverse coded)” Higher PRBI scores reflect endorsement of US school’s literacy beliefs, and research has shown that Mexican-American women who hold beliefs consistent with the schools’ literacy beliefs engage more frequently in literacy practices [28, 29].

#### 2.4.2. Demographics.

Parent participants completed a demographic survey in Spanish that included date of birth of parent and child, gender, highest parental education level, and country of origin of parent and child. Acculturation was measured using the widely used and previously validated Short Acculturation Scale for Hispanics (SASH) [30]. The SASH asks parents what language they speak, use at home, think in, and use among friends given the following options: Spanish only, Spanish better than English, Spanish and English equally, English better than Spanish, and English only. Score range is 1 to 5; <2.99 indicates a low level of acculturation. In our sample, the internal reliability of the SASH was *r* = 0.81.

Anthropomorphic measurements, psychosocial survey measures, and accelerometry were also collected at both baseline and followup but are not reported here.

## 3. Results

### 3.1. Sample Description.

The majority of our participants were mothers and first-generation Mexican immigrants with US-born children. Among parents, almost two-thirds (65%) had not completed high school or received a GED equivalent. Almost all participating adults (97%) had a low level of acculturation, even though the majority of them reported living in the US for 5–14 years. Eighty percent (80%) of participating parents were overweight/obese (BMI ≥ 25 kg/m^2^), and 42% of preschool-aged children were overweight/obese (BMI percentile ≥ 85%), which is markedly higher than the state averages for both groups, 68% and 14%, respectively [31, 32].

Randomization was effective: there were no significant differences between treatment groups on the battery of demographics given at baseline. Table 1 shows baseline demographics for study participants with pre- and postintervention data.

### 3.2. Result of Randomization on Dependent Variable.

To confirm that randomization resulted in equivalent groups on the dependent variable of interest, we compared verbal participation scores across treatment groups at baseline. Table 2 shows that the distribution of the verbal participation variable was equivalent at baseline across the two groups. These analyses were run for all 106 participants at baseline (not reported) and again for the 79 families who had both baseline and follow-up data on the outcome of interest. Results did not vary, suggesting that participant attrition did not bias results on the outcome variable.

### 3.3. Regressions.

Because assignment to the experimental condition was random, a regression of postintervention scores on group was conducted to reveal the causal effect of group on the dependent variable. Results of the regression analysis predicting follow-up verbal participation using group assignment and preintervention verbal participation are presented in Table 3.

Group assignment had a significant effect on postintervention verbal participation, such that participants in the bilingual early language development intervention scored higher compared to those assigned to the healthy lifestyle intervention (*P* = .016). R-squared indicated that the model explained 31% of the variance. Effect size was Cohen’s *d* = 0.46. We can consider this effect size “medium” based on traditional interpretation [33], small/medium/large *~* 0.2/0.5/0.8.

The baseline verbal participation score had, as expected, explanatory power for the follow-up score. We also run a regression that included, beside the group and the preintervention verbal participation, also child age, child gender, and parent age (not reported). However, all three were nonsignificant.

## 4. Discussion

The results of this study showed that parental involvement in a brief bilingual literacy intervention based in a community setting is effective in improving the value Latino immigrant parents place on their children’s active participation in joint reading over a 3-month period. Building on previous research, this study suggests that educational outcomes may be improved by educating parents on the value of playful conversations over books in one’s native language and providing parents with the simple tools they need to actively engage in the learning and teaching processes of their children. The results of this study have implications for both educators and policymakers looking for brief, low-cost interventions that have the potential to impact parental involvement in the cognitive and verbal development of their children and ultimately to improve educational outcomes for Latino students in the United States.

The majority of participating parents did not have a high school diploma or equivalent and were low acculturated. This intervention provides an opportunity to involve parents in the early childhood education of their child regardless of literacy and English-language proficiency skills. The majority of immigrant parents, regardless of the level of education, display willingness to participate in their child’s education but did not necessarily know the steps to take [15]. Despite limited English proficiency, these parents were able to participate in interactive reading activities with their child and demonstrate an increase in verbal participation. This is of note because limited English proficiency demonstrated by both parents has been associated with a drastic decrease in the English proficiency of the child [3]. By providing an intervention that can be used in a bilingual setting, non-English-speaking parents are given an important tool for participation in their child’s early education which in turn gives the child better education outcomes. Studies suggest that early encounters with literacy in any language will foster later literacy development [34]. Efforts to improve school readiness are most effective when they address the capacity of families and communities to provide developmentally enhancing opportunities for their young children [35].

The low cost and brief time span of this intervention offer meaningful policy implications for the future. This intervention could be useful for educators in areas heavily populated with Latino students and can be delivered in a local community center and/or branch library to make it accessible. In the US, city-operated community centers are often located in the centers of neighborhoods yet are often underutilized by the larger community. Three 60-minute monthly sessions focused on existing parental strengths are markedly less time consuming than sessions designed to teach parents how to read or speak English, and thus, this approach has the potential to be less intimidating, less bur-densome, and thus more impactful within this community. As mentioned previously, Latinos are less likely than children from any other racial or ethnic group to be enrolled in an early childhood program [6]. Thus, this would be a vehicle for providing to parents of children who do not attend structured early educational programming the skills they need to facilitate their children’s early language development.

### 4.1. Limitations.

A single interventionist delivered both the healthy lifestyle and the literacy intervention, allowing for the potential of contamination. However, a treatment fidelity plan was followed and fidelity of both conditions was assessed three times through the study period and indicated high fidelity of treatment implementation for both arms of the study. Most, importantly, at none of the monitored healthy lifestyle intervention sessions was information from the literacy intervention conveyed to participants.

There was some participant attrition over the course of the study. However, it does not seem to have been related to the dependent variable of interest. While the distribution of the verbal participation scores did not change when we restricted data analysis to only those parents with longitudinal data, this fact does not guarantee that attrition could not have affected our estimates. Indeed, the subsample of families with longitudinal data could have been different from the sample of baseline families according to unobserved dimensions that may be relevant for posttreatment outcome. For Example, two families may have the same baseline verbal participation scores but one family could have a higher willingness to improve the reading abilities of their child. This could have biased our estimates.

### 4.2. Conclusion.

According to Eamon, “Interventions that assist Latino parents in providing cognitively stimulating home environments, in being involved with the student’s academic life … can increase Latino youth academic achievement” [10]. This brief intervention assists Latino parents in providing cognitively stimulating home environments for their preschool-aged children by altering the way parents engage in conversation with their children using their native language and books. It addresses many of the well-documented barriers to reduced parental participation in their children’s later academic achievement: limited English-language skills, transportation, and busy, inflexible work schedules. It fills the existing gap in parent-implemented, strength-based approaches to improving early childhood literacy. Altering parental attitudes on the value placed on engaging in dialog with their children in their native language at an early age might empower Latino parents to become more involved in the education and academic achievement of their children.

## Figures and Tables

**Table 1:** Preintervention demographics of participating Latino parent-child dyads (*N* = 79).

Domain	Literacy intervention		Healthy lifestyles intervention		*P* value^1^
Child	Mean	S.D.	Mean	S.D.	
Age	4.1	0.9	4.2	0.9	0.85
Gender (% female)	54.6		45.7		1.00
Adult					
Age	32.3	5.7	30.7	6.0	0.22
Acculturation^2^	1.4	0.6	1.3	0.5	0.55
Relationship to child^3^	93%		94%		1.00
Maternal education^4^					0.75
<High school	64%		66%		
≥HS < college	27%		31%		
≥College	9%		3%		

1 *T*-tests were used except for categorical variables (those reported only in percentages) where exact Fisher tests were used.

2 Short Acculturation Scale for Hispanics (SASH).

3 Percentage of respondents who are mothers.

4 Percentage of adults by education category in each group.

**Table 2:** Group comparison on verbal participation preintervention (*N* = 79).

Group	*N*	Mean	Median	SD	Min	Max
Literacy intervention	44	3.30	3.30	.49	1.90	4.0
Healthy lifestyle intervention	35	3.33	3.20	.44	2.60	4.0
Total	79	3.31	3.30	.46	1.90	4.0

**Table 3:** Regression analysis predicting verbal participation postintervention scores.

	Beta	SE	*t*	*P*	95% CI	
Group	−.19	.08	−2.47	.016	−.34	−.04
Verbal participation preintervention	.46	.08	5.46	.000	.29	.62
Intercept	1.96	.28	6.99	.000	1.40	2.52

## Appendix Parent Reading Belief Inventory: Verbal Participation

The next questions are about reading to this child. Indicate how much you agree or disagree with the following statements.

1. When we read I try to sound excited so my child stays interested.
  - Strongly Disagree
  - Disagree
  - Agree
  - Strongly Agree
2. Children learn new words, colors, names, and so forth from books.
  - Strongly Disagree
  - Disagree
  - Agree
  - Strongly Agree
3. Reading helps children be better talkers and better listeners.
  - Strongly Disagree
  - Disagree
  - Agree
  - Strongly Agree
4. My child knows the names of many things he or she has seen in books.
  - Strongly Disagree
  - Disagree
  - Agree
  - Strongly Agree
5. I find it boring or difficult to read to my child.
  - Strongly Disagree
  - Disagree
  - Agree
  - Strongly Agree
6. When we read, I want my child to help me tell the story.
  - Strongly Disagree
  - Disagree
  - Agree
  - Strongly Agree
7. I ask my child a lot of questions when we read.
  - Strongly Disagree
  - Disagree
  - Agree
  - Strongly Agree
8. When we read, I want my child to ask questions about the book.
  - Strongly Disagree
  - Disagree
  - Agree
  - Strongly Agree
9. When we read we talk about the pictures as much as we read the story.
  - Strongly Disagree
  - Disagree
  - Agree
  - Strongly Agree

## References

[1] U.S. Department of Education, “Trends in High School Dropout and Completion Rates in the United States: 1972–2009,” Compendium Report, 2011, http://nces.ed.gov/pubs2012/2012006.pdf.

[2] Ceballo R, “From barrios to yale: the role of parenting strategies in Latino families,” Hispanic Journal of Behavioral Sciences, vol. 26, no. 2, pp. 171–186, 2004.

[3] Lahaie C, “School readiness of children of immigrants: does parental involvement play a role?” Social Science Quarterly, vol. 89, no. 3, pp. 684–705, 2008.

[4] Araújo Dawson B and Williams SA, “The impact of language status as an acculturative stressor on internalizing and externalizing behaviors among Latino/a children: a longitudinal analysis from school entry through third grade,” Journal of Youth and Adolescence, vol. 37, no. 4, pp. 399–411, 2008.

[5] De Feyter JJ and Winsler A, “The early developmental competencies and school readiness of low-income, immigrant children: influences of generation, race/ethnicity, and national origins,” Early Childhood Research Quarterly, vol. 24, no. 4, pp. 411–431, 2009.

[6] U.S. Department of Education National Center for Educational Statistics, Statistics in Brief-March 2000: Home Literacy Activities and Signs of Children’s Emerging Literacy, 1993–1999, U.S. Government Printing Office, Washington, DC, USA, 2000.

[7] NICHD Early Child Care Research Network, Child Care and Development: Results from the NICHD Study of Early Child Care and Youth Development, Guilford, New York, NY, USA, 2005.

[8] Carreón GP, Drake C, and Barton AC, “The importance of presence: immigrant parents’ school engagement experiences,” American Educational Research Journal, vol. 42, no. 3, pp. 465–498, 2005.

[9] Plunkett SW, Behnke AO, Sands T, and Choi BY, “Adolescents’ reports of parental engagement and academic achievement in immigrant families,” Journal of Youth and Adolescence, vol. 38, no. 2, pp. 257–268, 2009. 19636722 10.1007/s10964-008-9325-4

[10] Eamon MK, “Social-demographic, school, neighborhood, and parenting influences on the academic achievement of latino young adolescents,” Journal of Youth and Adolescence, vol. 34, no. 2, pp. 163–174, 2005.

[11] Kuperminc GP, Darnell AJ, and Alvarez-Jimenez A, “Parent involvement in the academic adjustment of Latino middle and high school youth: teacher expectations and school belonging as mediators,” Journal of Adolescence, vol. 31, no. 4, pp. 469–483, 2008. 17953983 10.1016/j.adolescence.2007.09.003

[12] Turney K and Kao G, “Barriers to school involvement: are immigrant parents disadvantaged?” Journal of Educational Research, vol. 102, no. 4, pp. 257–271, 2009.

[13] Schaller A, Rocha LO, and Barshinger D, “Maternal attitudes and parent education: how immigrant mothers support their child’s education despite their own low levels of education,” Early Childhood Education Journal, vol. 34, no. 5, pp. 351–356, 2007.

[14] Mushi SLP, “Acquisition of multiple languages among children of immigrant families: parents’ role in the home-school language pendulum,” Early Child Development and Care, vol. 172, pp. 517–530, 2002.

[15] Moreno R and Lopez JA, Latina Mothers’ Involvement in Their Children’s Schooling: The Role of Maternal Education and Acculturation, JSRI Working Paper no. 44. JSRI Research & Publications Working Paper Series, 1999.

[16] Luo L, Luk G, and Bialystok E, “Effect of language proficiency and executive control on verbal fluency performance in bilinguals,” Cognition, vol. 114, no. 1, pp. 29–41, 2010. 19793584 10.1016/j.cognition.2009.08.014

[17] Lisette M, “Language proficiency in bilingual preschoolers and its relationship to academic achievement,” Disseration Abstracts International Section A: Humanities and Social Sciences, 70(9-A)(3313), 2009.

[18] Roberts TA, “Home storybook reading in primary or second language with preschool children: evidence of equal effectiveness for second-language vocabulary acquisition,” Reading Research Quarterly, vol. 43, no. 2, pp. 103–130, 2008.

[19] Minkler M and Wallerstein N, Community-Based Participatory Research for Health, Jossey-Bass, San Francisco, Calif, USA, 2003.

[20] Alvarez RA, Vasquez E, Mayorga CC, Feaster DJ, and Mitrani VB, “Increasing minority research participation through community organization outreach,” Western Journal of Nursing Research, vol. 28, no. 5, pp. 541–560, 2006. 16829637 10.1177/0193945906287215 PMC2536763

[21] Eakin EG, Bull SS, Riley K, Reeves MM, Gutierrez S, and McLaughlin P, “Recruitment and retention of Latinos in a primary care-based physical activity and diet trial: the Resources for Health study,” Health Education Research, vol. 22, no. 3, pp. 361–371, 2007. 16963726 10.1093/her/cyl095

[22] Keller CS, Gonzales A, and Fleuriet KJ, “Retention of minority participants in clinical research studies,” Western Journal of Nursing Research, vol. 27, no. 3, pp. 292–306, 2005. 15781904 10.1177/0193945904270301

[23] McQuiston C and Uribe L, “Latino recruitment and retention strategies: community-based HIV prevention,” Journal of Immigrant Health, vol. 3, no. 2, pp. 97–105, 2001. 16228793 10.1023/A:1009565900783

[24] Washington Learning Systems, Language is the Key: A Program for Building Language and Literacy in Early Childhood, Washington Learning Systems, Seattle, Wash, USA, 2006, http://www.edgateway.net/cs/es/view/lwe/93615/.

[25] Dale PS, Crain-Thoreson C, Notari-Syverson A, and Cole K, “Parent-child book reading as an intervention technique for young children with language delays,” Topics in Early Childhood Special Education, vol. 16, no. 2, pp. 213–235, 1996.

[26] Lim YS and Cole KN, “Facilitating first language development in young Korean children through parent training in picture book interactions,” Bilingual Research Journal, vol. 26, no. 2, pp. 367–381, 2002.

[27] Bellg AJ, Resnick B, Minicucci DS , “Enhancing treatment fidelity in health behavior change studies: best practices and recommendations from the NIH Behavior Change Consortium,” Health Psychology, vol. 23, no. 5, pp. 443–451, 2004. 15367063 10.1037/0278-6133.23.5.443

[28] DeBaryshe BD and Binder JC, “Parent reading belief inventory,” Perceptual and Motor Skills, vol. 78, no. 3, pp. 1303–1311, 1994.

[29] Rodriguez BL, Hammer CS, and Lawrence FR, “Parent reading belief inventory: reliability and validity with a sample of Mexican American mothers,” Early Education and Development, vol. 20, no. 5, pp. 826–844, 2009. 23293507 10.1080/10409280802581276 PMC3536018

[30] Barona A and Miller JA, “Short acculturation scale for Hispanic youth (SASHY): a preliminary report,” Hispanic Journal of Behavioral Sciences, vol. 16, pp. 155–162, 1994.

[31] Trust for America’s Health, F as in Fat: How Obesity Policies are Failing in America 2010, Robert Woord Johnson Foundation, 2010.

[32] Centers for Disease Control and Prevention, “Obesity prevalence among low-income, preschool-aged children—United States, 1998–2008,” Morbidity and Mortality Weekly Report, vol. 58, no. 28, pp. 769–773, 2009. 19629026

[33] Agresti A, “Modelling patterns of agreement and disagreement,” Statistical methods in medical research, vol. 1, no. 2, pp. 201–218, 1992. 1341658 10.1177/096228029200100205

[34] Reese L, Garnier H, and Gallimore R, “Longitudinal analysis of the antecedents of emergent Spanish literacy and middle-school English reading achievement of Spanish-speaking students,” American Educational Research Journal, vol. 37, no. 3, pp. 633–662, 2000.

[35] Gonzalez JE and Uhing BM, “Home literacy environments and young Hispanic children’s English and Spanish oral language: a communality analysis,” Journal of Early Intervention, vol. 30, no. 2, pp. 116–139, 2008.

